# Retraction: Minimally Invasive Approaches for High-Risk and Elderly Patients With Acute Cholecystitis: A Systematic Review of Techniques and Outcomes

**DOI:** 10.7759/cureus.r174

**Published:** 2025-04-01

**Authors:** Mark Abdelmaseeh, Asem Mhmndar, Ayesha Siddiqa, Shoaib S Ahamed, Kanwal Sheraz, Ahmad F Mohd, Haider Ali, Abdullah Shehryar, Abdur Rehman, Khakan Murtaza

**Affiliations:** 1 General Surgery, Assiut University, Assiut, EGY; 2 Internal Medicine, Thumbay University Hospital, Ajman, ARE; 3 Internal Medicine, Rehman Medical Institute, Peshawar, PAK; 4 Gastroenterology, Nottingham University Hospitals NHS Trust, Nottingham, GBR; 5 General Surgery, Mayo Hospital, Lahore, PAK; 6 Internal Medicine, Allama Iqbal Medical College, Lahore, PAK; 7 Surgery, Mayo Hospital, Lahore, PAK; 8 Internal Medicine, Nishtar Medical University, Multan, PAK

The Editors-in-Chief have retracted this article. An investigation by the journal found evidence of authorship manipulation. The Editors-in-Chief therefore no longer have confidence in the provenance and reliability of the article contents. The authors agree with the decision to retract.

